# Augmentation colocystoplasty and incontinence surgery in patients with spina bifida – our ongoing experience

**DOI:** 10.1186/1743-8454-7-S1-S7

**Published:** 2010-12-15

**Authors:** Prashanth Kumar, Anant Bangar, Janani Krishnan, Santosh Karmarkar

**Affiliations:** 1 Department of Pediatric Surgery, Lilavati Hospital and Research Centre, Bandra Reclamation, Bandra (W) Mumbai-400050, India

## Background

Successful management of urinary incontinence is a cornerstone in endowing patients of neurogenic incontinence with a good quality of life. In recent times introduction of clean intermittent catheterization and anticholinergics at an early age is thought to avoid future incontinence surgery. Over the last few years, we have a growing experience in the management of urinary incontinence in spina bifida. In this paper we present, a cohort of 9 consecutive cases of colocystoplasty and/or bladder neck repair and Mitrofanoff and/or MACE procedures. A thorough preoperative work up, proper selection of cases, protocol based intraoperative and postoperative management is required for obtaining good results. These aspects are discussed in this paper.

## Materials and methods

We present 9 of our patients in the age group of 4yrs-19yrs, out of which 5 were boys and 4 girls. Augmentation colocystoplasty was done for all of them,7 underwent Mitrofanoff,5 underwent MACE,4 patients had a wide bladder neck and DEFLUX was injected in 2 of these patients,1 underwent Young-Dees-Leadbetter repair and a bladder neck ventrisuspension was done for 1 patient. Preoperative preparation extending over six months to one year included careful evaluation of each patient, thorough investigations, CIC training, anticholinergic medication, parental counselling and meticulous documentation.

## Results

The dry interval of our patients preoperatively ranged from <10mins to 120 mins(mean of 45 mins) which improved to a mean dry interval of 4 hours(range of 3 to 7 hrs) postoperatively .Young-Dees-Leadbetter and bladder neck hitch showed good results, whereas results of periurethral injection of DEFLUX were not encouraging. The Mitrofanoff and MACE stomas functioned well. There were no major complications in any of our patients postoperatively.

## Conclusions

With careful preoperative evaluation and urinary work up beginning 6months to 1year prior to the proposed date of surgery, proper CIC training, anticholinergic medication and meticulous augmentation technique, most patients with neurogenic incontinence stand to benefit significantly by augmentation colocystoplasty. Easy access continent channels such as a Mitrofanoff makes CIC a simpler task. We recommend augmentation colocystoplasty with adjunct procedures to achieve social continence in patients with neurogenic incontinence.

